# Elevated CO_2_ enhances aerobic scope of a coral reef fish

**DOI:** 10.1093/conphys/cot023

**Published:** 2013-09-21

**Authors:** Jodie L. Rummer, Jonathan A. W. Stecyk, Christine S. Couturier, Sue-Ann Watson, Göran E. Nilsson, Philip L. Munday

**Affiliations:** 1 ARC Centre of Excellence for Coral Reef Studies, James Cook University, Townsville, QLD 4811, Australia; 2 Programme for Physiology and Neurobiology, Department of Biosciences, University of Oslo, 0316 Oslo, Norway; 3 Department of Biological Sciences, University of Alaska Anchorage, Anchorage, AK 99508, USA; 4 School of Marine and Tropical Biology, James Cook University, Townsville, QLD 4811, Australia

**Keywords:** aerobic scope, climate change, coral reef fish, ocean acidification

## Abstract

The uptake of anthropogenic CO_2_ by the ocean has been suggested to impact marine ecosystems by decreasing the respiratory capacity of fish and other water breathers. We investigated the aerobic metabolic scope of the spiny damselfish, *Acanthochromis polyacanthus*, from the Great Barrier Reef, Australia when exposed for 17 days to CO_2_ conditions predicted for the end of the century (946 μatm CO_2_). Surprisingly, resting O_2_ consumption rates were significantly lower and maximal O_2_ consumption rates significantly higher in high-CO_2_-exposed fish compared with control fish (451 μatm CO_2_). Consequently, high-CO_2_-exposed fish exhibited an unexpected increase in absolute (38%) and factorial aerobic scopes (47%). Haematological and muscle water changes associated with exercise were not affected by CO_2_ treatment. Thus, contrary to predictions, our results suggest that elevated CO_2_ may enhance aerobic scope of some fish species. Long-term experiments are now required to assess the response to elevated CO_2_ further, because developmental and transgenerational effects can be dramatic in fish. Ultimately, understanding the variability among species regarding the effects of CO_2_ on aerobic scope will be critical in predicting the impacts of ocean acidification on marine communities and ecosystems.

## Abstract (precis)

The oceans are absorbing excess atmospheric CO_2_, and this is causing ocean acidification. Surprisingly, one coral reef damselfish exhibits enhanced aerobic performance after living at projected future ocean CO_2_ levels for 17 days. Identifying both the winners and losers under climate change scenarios is vital to conserving marine biodiversity.

## Introduction

Atmospheric CO_2_ levels are rising, leading to a corresponding increase in CO_2_ and a decrease in pH at the ocean surface, a process known as ocean acidification (Doney, 2010). Future CO_2_ levels are expected to impact marine ecosystems widely, because the scope for aerobic performance in fish and other water breathers is predicted to decrease at higher CO_2_ levels (Pörtner and Farrell, 2008). Reductions in aerobic scope (the difference between resting and maximal oxygen consumption rates) result in less energy being available for life-history processes, such as growth and reproduction (Donelson *et al.*, 2010; Pörtner and Peck, 2010). Thus, understanding how elevated CO_2_ influences aerobic scope is important for predicting the ecological impacts of ocean acidification on marine ecosystems (Ishimatsu *et al.*, 2008; Pörtner and Farrell, 2008; Munday *et al.*, 2012).

Consistent with theoretical predictions (Pörtner and Farrell, 2008), reduced aerobic scope at near-future CO_2_ levels (∼1000 μatm) has been demonstrated in two coral reef cardinalfishes (*Ostorhinchus doederleini* and *Ostorhinchus cyanosoma*; Munday *et al.*, 2009). The negative effects of elevated CO_2_ on cardinalfishes may be attributed to the fish living in tropical waters near the upper end of their thermal range, as they are particularly temperature-sensitive species (Gardiner *et al.*, 2010). However, in other tropical fishes, near-future CO_2_ levels appear to have a beneficial or hormetic effect on aerobic performance and life-history traits (Miller *et al.*, 2013; Couturier *et al.*, 2013). Recent studies have even demonstrated a mechanistic basis for enhanced oxygen delivery to tissues in the presence of low levels of hypercapnia that is unique to teleost fishes (Rummer and Brauner, 2011; Rummer *et al.*, 2013), but it is unknown how widespread the phenomenon may be. Moreover, at even higher CO_2_ levels the benefits may disappear, as indicated by research on Atlantic cod (*Gadus morhua*), in which no changes in oxygen consumption rates were observed at CO_2_ levels several times higher than end-of-century predictions (Melzner *et al.*, 2009). Likewise, Couturier *et al.* reported that juvenile damselfish (*Pomacentrus amobinensis*) no longer exhibited a higher maximal aerobic capacity at CO_2_ levels only moderately higher than end-of-century predictions (at ∼1400–2400 μatm; Couturier *et al.*, 2013). In fact, most of the earlier studies on fish exposed to CO_2_ levels 5–50 times greater than end-of-century predictions demonstrated no effect on performance (McKenzie *et al.*, 2003; Deigweiher *et al.*, 2008; Ishimatsu *et al.*, 2008; Baker *et al.*, 2009; reviewed by Brauner and Baker, 2009). Given that the physiological responses observed in fish at near-future CO_2_ levels (<1000 μatm) may be different from responses at the extreme CO_2_ levels often used in earlier studies (5000 to >50 000 μatm), it is important to test the effects of climate change-relevant CO_2_ levels on fish and other marine species to determine if they have generally negative effects, as predicted by theory, or potentially positive effects in some species, as suggested by recent experimental studies.

Habitat may also play an important role in determining the sensitivity of a species to increased CO_2_. Fluctuating CO_2_ levels are common in coastal marine ecosystems (Hofmann *et al.*, 2011), and coral reef ecosystems may already experience diurnal fluctuations in CO_2_ that reach or even exceed the average projected levels for the year 2100 (Melzner *et al.*, 2012; Shaw *et al.*, 2013). Therefore, some species may already show homeostatic adaptations to frequent CO_2_ fluctuations (McKenzie *et al.*, 2003; Ishimatsu *et al.*, 2008; Baker *et al.*, 2009; Brauner and Baker, 2009) that explain their ability to maintain performance at projected future CO_2_ levels. Given that increased uptake of CO_2_ by the ocean will affect both the average CO_2_ level and the magnitude of extreme CO_2_ levels, it is important to consider both physiological sensitivity and the habitat occupied in determining which species will exhibit positive and which species will exhibit negative responses to rising CO_2_ levels in the ocean.

We tested the effect of surface ocean CO_2_ levels projected for 2100 under Representative Concentrations Pathway 8.5 (RCP 8.5 = 936 μatm; Meinshausen *et al.*, 2011) on resting 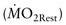 and maximal O_2_ consumption rates 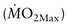 to calculate aerobic scope for the spiny damselfish, *Acanthochromis polyacanthus*, a model species for studying climate change impacts on reef fishes (Nilsson *et al.*, 2007, 2009; Donelson *et al.*, 2012). In addition to our primary aim, we also measured key haematological and tissue variables to examine the physiological status of CO_2_-exposed fish immediately following exercise to provide insight into the physiological mechanisms that may underlie the effects of CO_2_ on aerobic performance.

## Materials and methods

### Experimental animals

*Acanthochromis polyacanthus* (standard length, 63.5 ± 1.0 mm; wet mass, 11.41 ± 0.78 g; means ± SD) were collected from the Lizard Island lagoon (14°40′08′′S; 145°27′34′′E), Great Barrier Reef, Australia and maintained in the laboratory in a flow-through seawater system at ambient summer temperatures (27.3–30.6**°**C) for ∼14 days prior to CO_2_ treatment. Fish were then randomly removed from holding aquaria and evenly distributed among four 35 l aquaria supplied with seawater at present-day control CO_2_ levels (451 μatm) and four with high-CO_2_ water (946 μatm; Table 1). Fish were kept in CO_2_ treatments for 17 days and fed to satiation twice daily (NRD pellets; INVE Aquaculture, Salt Lake City, UT, USA), but food was withheld for 24 h prior to sampling or respirometry. All collection, care, and experimental protocols complied with James Cook University Animal Ethics Committee regulations (permit: #A1722).

**Table 1:** Mean seawater data (±SEM) and range for each treatment (values to nearest integer, one or two decimal places)

	Temperature (°C)			pH_NBS_			
Treatment	Mean	Range	Salinity (p.p.t.)	Mean	Range	Total alkalinity (μmol kg seawater^−1^)	Partial pressure of CO_2_ (μatm)
Control	29.2 (±0.1)	27.3–30.6	34.5	8.14 (±0.01)	8.11–8.17	2272 (±13)	451 (±16)
High CO_2_	29.3 (±0.1)	27.5–30.3	34.5	7.87 (±0.01)	7.84–7.89	2258 (±5)	946 (±29)

### Carbon dioxide treatment

Aquaria were supplied with seawater at present-day control CO_2_ levels (451 μatm) or high-CO_2_-equilibrated seawater (946 μatm). High-CO_2_ seawater was achieved by CO_2_ dosing a 60 l header tank to a set pH_NBS_ (National Bureau of Standards) to match the surface ocean CO_2_ level projected for 2100 under RCP 8.5 (Meinshausen *et al.*, 2011). A pH controller (Aqua Medic GmbH, Bissendorf, Germany) delivered a steady stream of CO_2_ into a powerhead in the bottom of the header tank if the pH rose above the set point. The pH was monitored regularly to ensure that it remained within ±0.05 of desired levels. Individual aquaria received CO_2_-equilibrated seawater from the 60 l header tank at ∼500 ml min^−1^. Control aquaria received seawater from a 60 l header tank diffused with ambient air. The temperature in each aquarium was measured twice daily. Seawater total alkalinity and pH_NBS_ for CO_2_ calculations were measured from replicate water samples of control and high-CO_2_ water taken at the start and end of the experiment. Total alkalinity was estimated by Gran titration using certified reference materials (Dr A. G. Dickson, Scripps Institution of Oceanography). Average seawater partial pressure of CO_2_ (*p*CO_2_) was calculated using these parameters in CO2SYS (Pierrot *et al.*, 2006) using constants from Dickson and Millero (1987) (Table 1).

### Resting and maximal oxygen consumption

Intermittent-flow respirometry has been found to provide a reliable estimate of standard or resting metabolic rates (Roche *et al.*, 2013) and was therefore used to determine resting O_2_ consumption rates for eight control and eight high-CO_2_-exposed fish in CO_2_ conditions. Fish were placed individually into 1615 ml, darkened respirometry chambers submerged in a temperature-controlled aquarium (29**°**C) and allowed 90 min to habituate to the chamber. Submersible pumps supplied a constant water flow (900 l h^−1^) from the aquaria through the chambers. In preliminary experiments, we determined that 90 min was ample time to ensure that O_2_ consumption rates had reached the lowest possible values, after which O_2_ consumption rates did not vary significantly. Thus, at 90 min, the water flow to each chamber was stopped for 15 min every 30 min over a period of 90 min (Supplementary material, Fig. S1). The time for which the water flow was interrupted was short enough to ensure that O_2_ did not fall below 80% saturation. The temperature-compensated O_2_ concentration (in milligrams per litre) of the water within each chamber was continuously recorded (1 s^−1^) using oxygen-sensitive REDFLASH dye on contactless spots (2 mm) adhered to the inside of each chamber and linked to a Firesting Optical Oxygen Meter (Pyro Science e. K., Aachen, Germany) via fibre-optic cables. Data were analysed using LabChart version 6.1.3 (ADInstruments, Colorado Springs, CO, USA). The value of 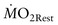 (in milligrams per kilogram per hour) was calculated from the average of the three slopes of O_2_ concentration (Supplementary material, Fig. S1), minus the background O_2_ consumption, which was measured daily before and at the end of each trial (assumed linear) and did not exceed 5% of the 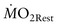 of the fish.

Following the measurement of 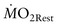, fish were held in individual mesh baskets for 1 h and fed *ad libitum* to boost O_2_ consumption further. The maximal O_2_ consumption rate was then determined in a circular swim respirometer (Nilsson *et al.*, 2007). To determine maximal oxygen consumption rates, fish were placed individually into a 1612 ml sealed vertical cylinder submerged in a temperature-controlled aquarium (29**°**C). A water current within the cylinder was created using a magnetic stirring bar and plate (below the cylinder), and the water speed was increased to the maximal speed at which the fish could sustain a steady position (see Nilsson *et al.*, 2007 for a diagram and a detailed description of the set-up). Criteria for obtaining the maximal sustained swimming speed at which 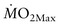 could be determined were that the fish had to be swimming against the current using pectoral fins only while maintaining the same position in the cylinder. Increasing the speed of the water current would result in the fish losing position. The decrease in O_2_ concentration in the cylinder was monitored with an oxygen probe (WTW OXI 340i, Weilheim, Germany) for up to 7 min, during which time the rate of O_2_ decline was stable. Data were analysed offline, and 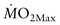 was calculated as described above for 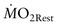. Absolute 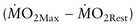 and factorial aerobic scopes 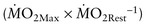 were calculated for each fish.

### Haematological and tissue analyses

Immediately following the measurment of 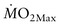, fish were euthanized by cranial concussion. The caudal fin was severed, blood was collected to analyse haemoglobin, glucose, and lactate concentrations, and epaxial muscle was dissected to calculate the percentage of water in the muscle. Additionally, eight control and eight high-CO_2_-exposed fish not subjected to respirometry were sampled to determine resting physiological status. Haemoglobin (Hb) concentration in blood was determined using 10 μl of whole blood and the HemoCue^®^ Hb 201 System, Australia Pty Ltd, Tumbi Umbi, NSW, Australia and reported as grams per 100 ml and millimolar haemoglobin tetramer (Hb_4_) using calibration curves previously verified on this species and according to Clark *et al.* (2008). Whole blood glucose and lactate concentrations (millimolar) were determined from two 15 μl samples using the Accutrend^®^ Plus (Roche Diagnostics Australia Pty Ltd Dee Why, NSW, Australia). Fulton's body condition factor (*K* = (*W* × 100) × *L*^−3^, where *W* is wet mass in grams and *L* is standard length in millimetres) was calculated to assess the length-to-weight ratio. The volume of plasma was insufficient for other analyses 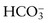 (e.g. Cl^−^,, total CO_2_, and catecholamines).

### Statistical analyses

Student's paired *t*-tests were used to compare 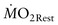 and 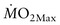 between control and high-CO_2_-exposed fish. Two-way ANOVAs and Holm–Sidak *post hoc* tests were used to compare haematological and tissue parameters between control and high-CO_2_-exposed fish at rest and post-exercise. Statistical analyses were conducted using SigmaPlot (Systat Software, Inc., Chicago, IL, USA).

## Results

**Figure 1:**
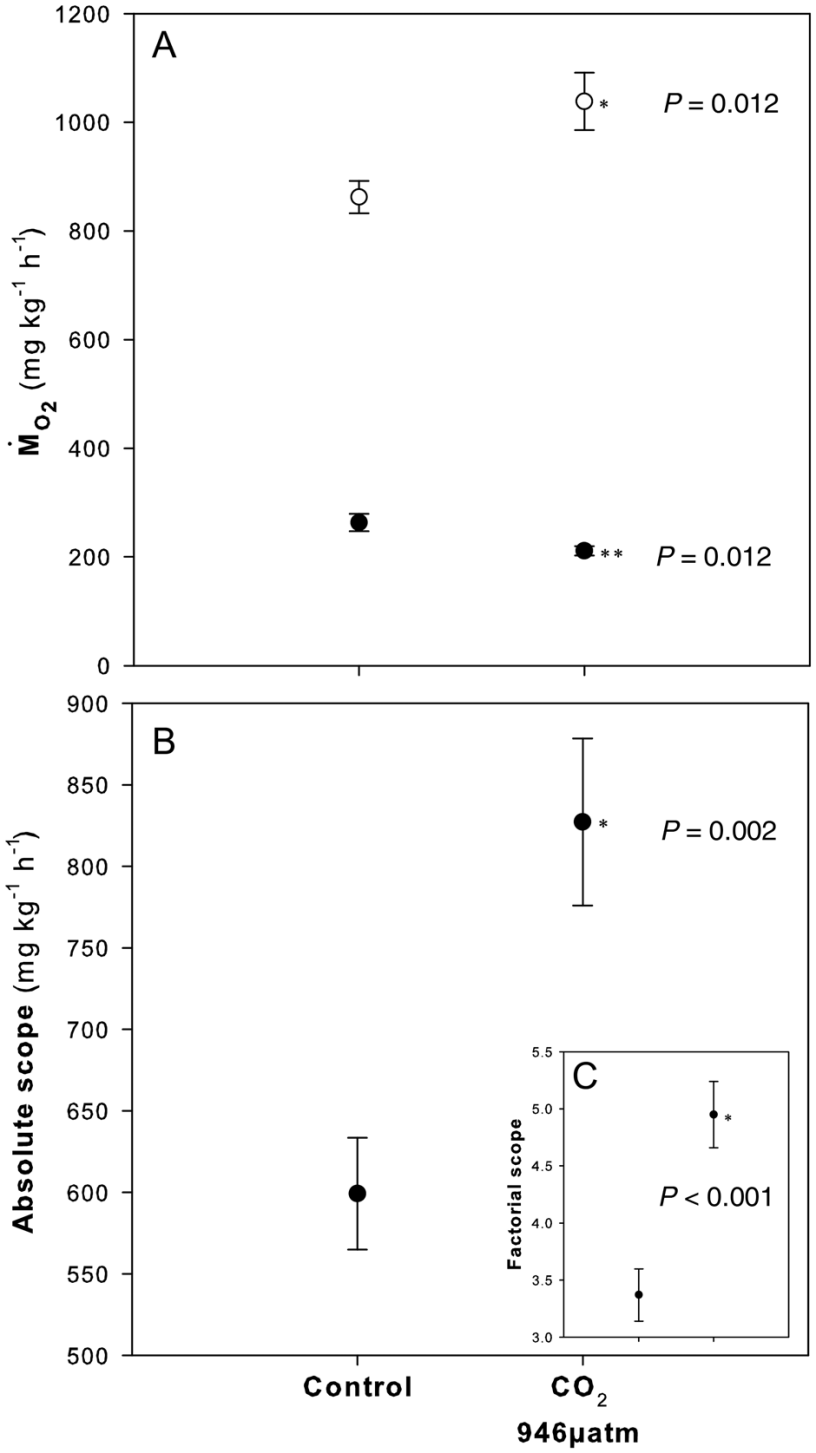
The effect of 17 days of exposure to high-CO_2_ on resting and maximal O_2_ consumption rates and absolute and factorial aerobic scope in spiny damselfish. (**A**) Resting (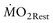; filled circles) and maximal oxygen consumption rates (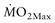; open circles). (**B**) Absolute aerobic scope 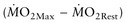. (**C**) Factorial aerobic scope 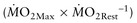. Values are means ± SEM. Asterisks demarcate significant differences from control values (Student's paired *t*-test).

The 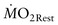 of high-CO_2_-exposed fish was ∼20% lower than that of control fish (*t*_(14)_ = 2.866, *P* = 0.012; Fig. 1A), whereas the 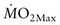 of high-CO_2_-exposed fish was ∼20% higher than that of control counterparts (*t*_(14)_ = −2.898, *P* = 0.012; Fig. 1A). Consequently, high-CO_2_-exposed fish exhibited a 38 and 47% higher absolute and factorial aerobic scope (*t*_(14)_ = −3.70, *P* = 0.002; Fig. 1B; and *t*_(14)_ = −4.29, *P* < 0.001; Fig. 1C).

Exposure to high-CO_2_ conditions for 17 days had no effect on the physiological parameters examined in resting conditions (Table 2 and Supplementary material, Table S1). Likewise, [Hb], [lactate], [glucose], and muscle water did not differ between control and CO_2_ treatment groups immediately following measurement of 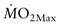. However, [lactate] and percentage muscle water increased following measurement of 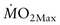, independent of CO_2_ treatment (*P* = 0.045 and *P* < 0.001, respectively). Body condition did not change in control or high-CO_2_ conditions (Table 2 and Supplementary material, Table S1). No significant interactions were detected between CO_2_ treatment and exercise (Supplementary material, Table S1).

**Table 2:** The effect of high CO_2_ and maximal swimming on body metrics, blood, and tissue variables of spiny damselfish

			Mass (g)	Standard length (mm)	Condition factor (*K*)	[Hb] (g 100 ml^−1^)	[Hb] (mM)	[Lactate] (mM)	[Glucose] (mM)	Muscle water (%)
Control	Rest	Mean	10.60	62.43	0.00424	6.50	1.03	1.33	2.87	74.25
		SEM	0.94	1.57	0.00008	0.30	0.05	0.30	0.40	0.41
		*n*	13	13	13	10	10	9	9	13
	Post-swimming	Mean	11.68	64.74	0.00445	7.20	1.14	3.27*	3.73	75.89*
		SEM	0.71	1.26	0.00011	0.31	0.05	1.20	0.49	0.28
		*n*	8	7	7	5	7	3	6	7
High CO_2_	Rest	Mean	13.20	65.35	0.00427	6.80	1.08	1.58	1.69	73.46
		SEM	0.37	2.69	0.00019	0.12	0.02	0.17	0.36	0.38
		*n*	10	10	10	7	5	9	9	10
	Post-swimming	Mean	10.17	61.59	0.00425	6.60	0.92	2.87*	2.67	75.00*
		SEM	1.02	2.17	0.00015	0.44	0.14	0.67	0.42	0.82
		*n*	8	8	8	8	8	7	7	8
Significance			n.s.	n.s.	n.s.	n.s.	n.s.	*P* = 0.045	n.s.	*P* < 0.001

Abbreviations: [Hb], haemoglobin concentration; n.s., non-significant. Asterisks demarcate significant differences between rest and post-swimming values within a given parameter; there were no effects of CO_2_ treatment or interaction between CO_2_ treatment and exercise (two-way ANOVA).

## Discussion

When exposed to CO_2_ levels relevant to end-of-century projections RCP 8.5 (Meinshausen *et al.*, 2011) for 17 days, spiny damselfish demonstrated an enhanced aerobic scope compared with control fish, contradicting predictions that elevated CO_2_ will reduce aerobic performance (Ishimatsu *et al.*, 2008; Pörtner and Farrell, 2008). The response differs from the 47% decrease in aerobic scope observed in coral reef cardinalfishes exposed to similar CO_2_ levels (Munday *et al.*, 2009), as well as the unchanged 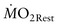 and 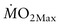 for other teleosts exposed to much higher CO_2_ levels (McKenzie *et al.*, 2003; Deigweiher *et al.*, 2008; Ishimatsu *et al.*, 2008; Melzner *et al.*, 2009). However, the response found in the present study is similar to the response of another damselfish, juvenile *Pomacentrus amboinensis*, which exhibited a 28–39% increase in 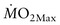 at similar CO_2_ levels (Couturier *et al.*, 2013). Parameters related to blood oxygen-carrying capacity, energy metabolism, and tissue hydration revealed the expected differences between fish at resting and post-swimming, but were not influenced by CO_2_ treatment. We discuss potential mechanisms for this unexpected enhancement of performance in conditions of elevated CO_2_.

### Resting oxygen consumption

Exposure to very high levels of CO_2_ (5000 to >50 000 μatm; 5–50 times higher than in present study) and the associated decrease in seawater pH induce hyperventilation and several physiological modifications at the fish gill, including increases in ion and acid–base regulation (Evans *et al.*, 2005; Brauner and Baker, 2009). Within the first 96 h of exposure to high CO_2_, fish elevate plasma [HCO_3_^−^] via equimolar decreases in [Cl^−^] to counter increased [H^+^] (Brauner and Baker, 2009; Esbaugh *et al.*, 2012). Ion exchange occurs largely at the gill, and when the main ion transporters operate at higher rates, rearrangements to energy budgets could result (Deigweiher *et al.*, 2010). Increased gill energy requirements (Deigweiher *et al.*, 2010) suggest that 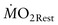 could increase during high-CO_2_ exposure, yet many studies have shown no change in 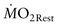 (Ishimatsu *et al.*, 2008; Couturier *et al.*, 2013). Furthermore, in the present study, we observed a decrease in 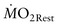 during high-CO_2_ exposure, suggesting decreased energy demands. A plasma acidosis is a characteristic response to elevated environmental CO_2_ (Claiborne *et al.*, 2002), but it is important to note that at the climate change-relevant CO_2_ levels (946 μatm) in this study, environmental *p*CO_2_ is still likely to be much lower than the plasma *p*CO_2_ of resting fish (Esbaugh *et al.*, 2012). The levels of hypercapnia used here still represent an outward, yet reduced, blood-to-environment CO_2_ gradient (Esbaugh *et al.*, 2012), but may not be problematic in comparison to higher levels examined in previous studies that would have severely impacted CO_2_ diffusion.

### Maximal oxygen consumption

Compared with control conditions, high-CO_2_-exposed spiny damselfish increased 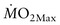. During maximal aerobic exercise, fish can increase functional respiratory surface areas by increasing gill blood perfusion, pressure, and lamellar recruitment (Wood and Randall, 1973; Evans *et al.*, 2005). While increasing gill surface area may satisfy increased O_2_ requirements, there may be a cost to osmoregulation (Randall *et al.*, 1972). Marine fish increase drinking rates to compensate for water loss over the gills, but may consequently expend more energy excreting excess ions across the gills. This did not appear to be the case in the present study. Here, exercised fish exhibited an increase in muscle water, which may indicate an increase in drinking; however, the response was uniform between control and CO_2_ treatment groups. Thus, although the upper limit to aerobic activity may be set by the need to defend ion balance (Gonzalez and McDonald, 1992), this critical threshold may not have been reached at the CO_2_ levels used in this study. Exercising spiny damselfish may be able to afford increases in the functional respiratory surface area of the gill, thereby boosting O_2_ uptake, but without significant ion and acid–base disturbances at these low levels of hypercapnia.

Whole blood lactate concentrations were also elevated in both control and CO_2_-treated fish post-exercise, with no effect of CO_2_. The finding suggests that both groups of fish were reaching roughly the same aerobic/anaerobic threshold and were potentially exerted to a similar extent. Nevertheless, the fish exposed to elevated CO_2_ for 17 days were able to do this while increasing O_2_ consumption rates. It may be that exposure to mild hypercapnia, as in this experiment, combined with the stress of exercise resulted in a release of catecholamines into the bloodstream, which has recently been demonstrated in rainbow trout to aid in increasing O_2_ uptake and potentially delivery (Rummer and Brauner, 2011; Rummer *et al.*, 2013). Clearly, more research is necessary to understand the mechanisms underpinning the increase in aerobic scope during mild hypercapnia observed here in the spiny damselfish.

### Significance and perspectives

Contrary to predicted physiological impacts of climate change (Pörtner and Farrell, 2008), aerobic scope of *A. polyacanthus* was increased upon exposure to predicted end-of-century CO_2_ levels. The finding adds to a growing number of studies showing that the effect of increased CO_2_ levels on aerobic performance varies dramatically among fish species, ranging from decreasing aerobic performance (Munday *et al.*, 2009) or no change in aerobic performance (McKenzie *et al.*, 2003; Deigweiher *et al.*, 2008; Ishimatsu *et al.*, 2008; Melzner *et al.*, 2009) to increasing aerobic performance (Couturier *et al.*, 2013). If aerobic scope underpins the performance of fish populations (Pörtner and Peck, 2010; Eliason *et al.,* 2011), ocean acidification could play an important role in altering the relative abundances of species, and thereby ecosystem dynamics and the structure of marine communities, especially in the face of fluctuating CO_2_ levels in coastal marine ecosystems. In the light of recent findings of strong developmental and transgenerational acclimation effects in fish exposed to elevated CO_2_ and/or temperature (Miller et al., 2012; Salinas and Munch, 2012; Scott and Johnston, 2012), it is even more important to understand this variable, perhaps species-specific response in aerobic scope that has important implications for the future structure of marine communities.

## Supplementary material

Supplementary material is available at *Conservation Physiology* online.

Supplementary Data
