# Supplementary material for: Elevated CO2 enhances aerobic scope of a coral reef fish
Source: Conserv Physiol. 2013 Sep 21;1(1):cot023. doi: 10.1093/conphys/cot023 (PMC4732439; doi:10.1093/conphys/cot023)
Supplement: Supplementary Data [file supp_1_1_cot023__index.html]

Elevated CO2 enhances aerobic scope of a coral reef fish — Supplementary Data 

# Elevated CO2 enhances aerobic scope of a coral reef fish

## Supplementary Data

Supplementary Data

**Files in this Data Supplement:**

- Supplementary Data - Docx file
