# Supplementary material for: Elevated CO2 enhances aerobic scope of a coral reef fish
Source: Conserv Physiol. 2013 Sep 21;1(1):cot023. doi: 10.1093/conphys/cot023 (PMC4732439; doi:10.1093/conphys/cot023)
Supplement: Supplementary Data [file supp_cot023_cot023supp.docx]

**Table S1:** Results from statistical analyses using two-way ANOVA

| **Source** | **df** | **MS** | **F** | ***P*** |
| --- | --- | --- | --- | --- |
| *Mass* | | | | |
| CO_2_ | 1 | 2.785 | 0.184 | 0.671 |
| Exercise | 1 | 8.912 | 0.589 | 0.448 |
| CO_2_ x Exercise | 1 | 39.538 | 2.612 | 0.115 |
| Residual | 35 | 15.139 |  |  |
| Total | 38 | 15.350 |  |  |
| *Standard length* | | | | |
| CO_2_ | 1 | 0.123 | 0.003 | 0.956 |
| Exercise | 1 | 4.744 | 0.118 | 0.733 |
| CO_2_ x Exercise | 1 | 82.632 | 2.059 | 0.160 |
| Residual | 34 | 40.135 |  |  |
| Total | 37 | 39.279 |  |  |
| *Condition factor* | | | | |
| CO_2_ | 1 | 9.03E-08 | 0.641 | 0.429 |
| Exercise | 1 | 6.20E-08 | 0.440 | 0.512 |
| CO_2_ x Exercise | 1 | 1.14E-07 | 0.809 | 0.375 |
| Residual | 34 | 1.41E-07 |  |  |
| Total | 37 | 1.38E-07 |  |  |
| *[Hb]* | | | | |
| CO_2_ | 1 | 0.148 | 0.184 | 0.672 |
| Exercise | 1 | 0.408 | 0.506 | 0.483 |
| CO_2_ x Exercise | 1 | 1.490 | 1.851 | 0.186 |
| Residual | 25 | 0.805 |  |  |
| Total | 28 | 0.801 |  |  |
| *[Lactate]* | | | | |
| CO_2_ | 1 | 2.204 | 1.307 | 0.263 |
| Exercise | 1 | 7.475 | 4.433 | 0.045 |
| CO_2_ x Exercise | 1 | 0.663 | 0.393 | 0.536 |
| Residual | 27 | 1.686 |  |  |
| Total | 30 | 1.868 |  |  |
| *[Glucose]* | | | | |
| CO_2_ | 1 | 6.526 | 4.236 | 0.049 |
| Exercise | 1 | 4.027 | 2.614 | 0.117 |
| CO_2_ x Exercise | 1 | 0.563 | 0.366 | 0.550 |
| Residual | 28 | 1.540 |  |  |
| Total | 31 | 1.769 |  |  |
| *Muscle water* | | | | |
| CO_2_ | 1 | 1.025 | 0.437 | 0.513 |
| Exercise | 1 | 39.221 | 16.714 | <0.001 |
| CO_2_ x Exercise | 1 | 1.792 | 0.764 | 0.388 |
| Residual | 34 | 2.347 |  |  |
| Total | 37 | 3.273 |  |  |

**Fig. S1:** Representative trace depicting the three slopes whereby the reduction in O_2_ concentration over a period of time was used to determine a resting O_2_ consumption rate for each animal.
